# Processing Stability of Carbon Nanofiber-Reinforced Glass Fiber/Polypropylene Composites Under Repeated Extrusion for Mechanical Recycling

**DOI:** 10.3390/ma18204777

**Published:** 2025-10-19

**Authors:** Tetsuo Takayama, Daisuke Shimizu, Shunsuke Kobayashi

**Affiliations:** 1 Graduate School of Organic Materials Science, Yamagata University, Yonezawa 992-8510, Japan; 2 Department of Polymeric and Organic Materials Engineering, Yamagata University, Yonezawa 992-8510, Japan

**Keywords:** carbon nanofiber (CBNF), circular economy, glass fiber-reinforced polypropylene, mechanical recycling, recyclable thermoplastics

## Abstract

Glass fiber-reinforced polypropylene (PP/GF) is used widely in lightweight automotive applications, but it is affected adversely by fiber breakage and matrix degradation during recycling. This study investigates the effects of carbon nanofiber (CBNF) addition on the recyclability of PP/GF composites subjected to repeated extrusion. Homo-type PP was compounded with GF and CBNFs and was processed for up to nine extrusion cycles. Melt viscosity, fiber morphology, flexural properties, interfacial shear strength, and notched Charpy impact strength were evaluated. Neat PP showed a pronounced increase in the melt volume-flow rate (MVR) with cumulative cycles, indicating molecular degradation. By contrast, CBNF-containing composites exhibited superior viscosity stability, with MVR increasing only 2.9-fold after nine cycles compared with 5.4-fold for GF-only systems. Fiber length was well maintained (96–98% retention). The flexural strength and modulus were preserved, respectively, as greater than 92% and 95%. The interfacial shear strength remained stable, whereas the impact strength decreased moderately but retained 84% of its initial value. These results underscore that a slight addition of CBNFs (5 wt%) suppresses viscosity loss effectively and stabilizes mechanical performance, offering a viable strategy for sustainable recycling of PP/GF composites in transportation applications.

## 1. Introduction

Global warming and resource depletion have become urgent global challenges, necessitating immediate countermeasures. In the transportation sector, particularly the automotive industry, reducing greenhouse gas (GHG) emissions through vehicle weight reduction is strongly demanded [1,2]. Fiber-reinforced thermoplastics (FRTPs) such as glass fiber-reinforced polypropylene (PP/GF) are applied widely as alternatives to steel and aluminum alloys because of their low density and high strength. These materials improve fuel efficiency and extend the driving range of electric vehicles. However, increasing amounts of production scrap and end-of-life components have necessitated the development of effective recycling technologies for a circular economy [3,4]. Mechanical recycling, which involves remelting and reprocessing, is regarded as particularly promising because of its energy efficiency and cost efficiency. Nevertheless, maintaining the mechanical performance of recycled composites persists as a major challenge [3,4].

The viability of mechanical recycling for FRTPs is a subject that has garnered significant interest, yet the extant literature on the subject is limited in scope and depth. A study of waste that includes fiber-reinforced plastics (FRPs) with thermosetting resins as the matrix material has revealed that approximately 95% of FRP and FRTP waste consists of glass fiber-reinforced materials [5]. In Europe, the annual generation of composite waste, including GFRP, is approximately 680,000 tons; however, the recycling processing capacity is estimated to be less than 100,000 tons [6]. Consequently, enhancing the recycling rate of plastics reinforced with glass fiber constitutes a pivotal component in the pursuit of a sustainable society.

It is generally known that the mechanical properties of PP/GF are superior to those of PP alone [7]. It is also known that these properties depend significantly not only on the nature of the polymer matrix but also on the length and orientation of the glass fibers [8]. Because glass fibers do not regenerate during recycling, breakage inevitably occurs, leading to property deterioration [8,9]. For example, Chen et al. reported that, in the context of mechanical recycling processes involving repeated extrusion and injection molding, glass fibers underwent fracturing due to the combined effects of shear stress and fiber-to-fiber collisions. This phenomenon led to a substantial decrease in the average fiber length. This decrease in fiber length directly leads to deterioration in tensile strength and impact strength, making it a primary cause of performance degradation in mechanical recycling [8].

To address this difficulty, several reinforcement strategies have been proposed, including hybrid fiber systems and nanofiller addition. Our earlier study demonstrated that dispersing carbon nanofibers (CBNFs) into aramid fiber-reinforced polypropylene improved the flexural modulus and strength to levels comparable to those of PP/GF [10]. Actually, CBNFs exhibit a high aspect ratio, excellent stiffness, and good interfacial compatibility with polypropylene. Furthermore, their whisker-like morphology provides high resistance to repeated melt compounding, suggesting that CBNFs are a promising reinforcement for improving recyclability. These findings indicate that CBNFs might suppress performance degradation associated with fiber length reduction in PP/GF composites.

However, systematic studies of PP/GF composites containing CBNFs under repeated extrusion are scarce. Particularly, neither the influence of recycling cycles on fiber length distribution, flexural, or impact properties, nor the role of CBNFs in mitigating property degradation has been clarified. Obtaining these findings is a necessary task for proposing measures to reduce performance degradation caused by mechanical recycling of PP/GF.

Conversely, the authors investigated the tensile properties of polypropylene repeatedly extruded using a twin screw extrusion method. It has been posited that an increase in the number of repeated extrusions alters the stereoregularity of PP, leading to a decrease in elastic modulus and yield stress [11]. This mechanism may also be applicable to PP/GF composites with dispersed glass fibers. Consequently, changes in the mechanical properties of PP/GF due to repeated extrusion are thought to be related not only to changes in fiber length but also to alterations in the mechanical properties of the matrix, polypropylene.

Therefore, this study was conducted to investigate the effects of CBNF dispersion on the physical and mechanical properties of PP/GF composites during repeated extrusion. Specifically, the melt viscosity, fiber length distribution and orientation, flexural strength, flexural modulus, and notched Charpy impact strength were evaluated.

The aim of this study is to enhance the mechanical properties and recyclability of PP/GF composites by developing and verifying the effectiveness of incorporating dispersed carbon nanofibers into the composites. The findings represent new insights into enhancing the mechanical recyclability of PP/GF composites and contribute to the development of sustainable polymer materials for circular economy applications.

## 2. Materials and Methods

The following experiments were conducted to achieve the objective of this study.

### 2.1. Materials

The present study aims to conduct trial production of PP/GF and PP/GF/CBNF. Polypropylene (PP, Novatec MA1B, Japan Polypropylene Corp., Tokyo, Japan) was used as the polymer matrix of this study. Density and Weight average molecular weight of PP is 0.9 g/cm^3^ and 312,000 g/mol [12], respectively.

Maleic anhydride-grafted polypropylene (MAH-PP, SCONA TSPP 10,213 GB; BYK, Chester, NY, USA) was employed as a compatibilizer. Density of MAH-PP is 0.9 g/cm^3^, and maleic anhydride content of MAH-PP is 2.0 wt.%. Chopped glass fibers (GF, T-351; Nippon Electric Glass Co., Ltd., Otsu, Japan) were used as reinforcement. According to the literature, GF has a density of 2.7 g/cm^3^. The reported fiber tensile strengths range from 1.7 GPa to 3.4 GPa, and the modulus of elasticity is 72 GPa [13]. The initial fiber length before compounding was 3 mm, with a 13 µm fiber diameter. Carbon nanofibers (CBNFs, ALP-NA1; Almedio Inc., Tokyo, Japan) were used as whisker-shaped nanofillers. Density of CBNF is 1.75 g/cm^3^. According to the extant literature, the tensile strength of CBNF is reported to be approximately 30 GPa, and its elastic modulus is approximately 700 GPa [14]. The CBNF diameter was 0.2–0.8 µm. Their initial length was 1–15 µm before compounding. These materials are new and do not utilize waste from the automotive industry.

### 2.2. Melt Compounding

The composite material was fabricated through a melt-compounding technique that incorporated twin-screw melt extrusion. Compounding was performed using a twin-screw extruder (15 mm screw diameter, L/D = 25, IMC0-00; Imoto Machinery Co., Ltd., Kyoto, Japan). The barrel temperature was set as 240 °C. The screw rotation speed was set as 75 rpm. The composites were prepared to have the following formulations: PP/MAH-PP/GF = 77/3/20 wt.%, PP/MAH-PP/GF/CBNF = 72/3/20/5 wt.%, and neat PP. The obtained strands were pelletized and subsequently subjected to repeated extrusion and molding processes.

### 2.3. Repeated Extrusion

In order to assess the mechanical recyclability of composite materials, a series of uniaxial melt extrusion tests was conducted, thereby generating a set of samples that exhibited varying degrees of molding history. Repeated melt processing was conducted using a single-screw extruder (16 mm screw diameter, L/D = 24, AS-1; Apex Co., Ltd., Saitama, Japan). The barrel temperature was maintained at 240 °C, with the screw speed set as 20 rpm. Samples were collected after 0, 2, 4, 6, 8, and 9 extrusion cycles. The amount required for injection molding produces about 40 specimens under each condition. Melt viscosity measurements were taken.

### 2.4. Melt Viscosity Measurement

In order to assess the impact of variations in molding history on melt viscosity, measurements were taken of the melt volume-flow rate (MVR). These measurements were used to determine the degree of apparent melt viscosity. It has been established that there is a negative correlation between this value and apparent viscosity [15]. The MVR was measured using a melt flow indexer (G-01; Toyo Seiki Seisaku-sho, Ltd., Tokyo, Japan) in accordance with ISO 1133 under conditions of 190 °C and a load of 2.160 kgf [15]. The MVR of each sample was evaluated. The number of tests was one for each condition. The reason for this is that the device used for this study can acquire MVR once every second during the test. A reliable average value is obtainable from a single test.

### 2.5. Injection Molding

**Figure 1:**
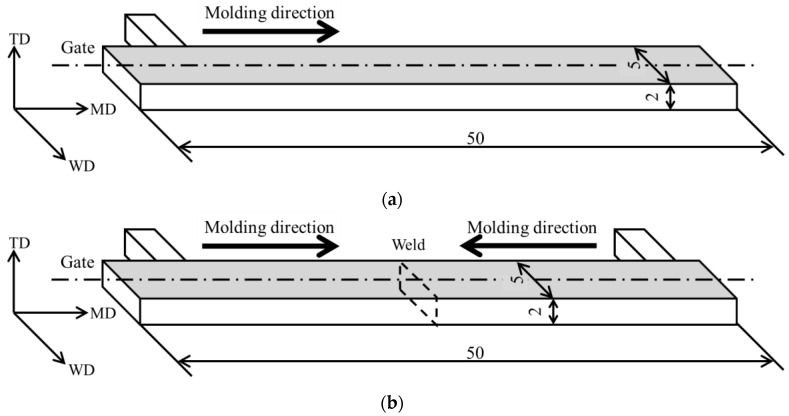
Specimen geometries of injection products: (**a**) single-gate and (**b**) double-gate. These figures were cited from [10]. The TD, WD, and MD in the figure indicate the thickness direction (TD), width direction (WD), and molding direction (MD), respectively.

Injection molding was performed on each sample to evaluate its mechanical properties. Test specimens were prepared using a microelectric injection molding machine (C, Mobile 0813; Shinko Sellbic Co., Ltd., Tokyo, Japan) under the molding conditions presented in Table 1. The molding parameters were fixed irrespective of the number of extrusion cycles. The specimen geometry is depicted in Figure 1, with dimensions of 50 mm (length), 2 mm (thickness), and 5 mm (width). The TD, WD, and MD in the figure indicate the thickness direction (TD), width direction (WD), and molding direction (MD), respectively. Both single-gate and double-gate molds were used. The latter was applied to form a weld line at the specimen center. In this study, 15 single-gate specimens and 20 double-gate specimens were fabricated.

### 2.6. Fiber Orientation and Fiber Length Analysis

The mechanical properties of glass fiber-reinforced composites are known to depend on the dispersion state and length of the glass fibers. X-ray Computed Tomography (CT) observation was performed to ascertain the orientation state and fiber length distribution of glass fibers within injection-molded parts. Fiber orientation and length distribution were analyzed using a microfocus X-ray CT system (ScanXmate-D225RSS270; Comscantecno Co., Ltd., Yokohama, Japan). The scanning conditions were the following: 60 kV tube voltage, 150 µA tube current, 9.0 W power, 25× magnification, and 5.08 µm/pixel resolution. The fiber orientation angle and its standard deviation were calculated from images of the skin and core layer regions of the molded product. In addition, average fiber length and its standard deviation were also calculated from images of the skin layer region. This analysis was performed using a single injection-molded product with a single gate under each condition.

### 2.7. Interfacial Mechanical Property Evaluation

The mechanical properties of glass fiber-reinforced composites are contingent not only on the aforementioned fiber orientation and fiber length distribution but also on the mechanical properties at the fiber-matrix interface. In this study, the interfacial shear strength was evaluated using a short beam shear test. Interfacial shear strength was evaluated using a tabletop universal testing machine (MCT-2150; A&D Co., Ltd., Tokyo, Japan) following the method described by Quang et al. [16]. The test conditions included a 10 mm/min loading speed and a 10 mm span length. Double-gate molded specimens were set in the WD and were bent until fracture at the weld line. The load–deflection curve was differentiated to obtain the stiffness–deflection curve. Two discontinuous drops in stiffness were identified. Using the loads found at these points, average shear stresses were calculated. Then the interfacial shear strength was obtained by synthesizing these stresses using the theorem of three squares [16]. This test was performed 10 times under each condition. The mean and standard deviation of the calculated interfacial shear strength were determined.

### 2.8. Flexural Property Evaluation

To assess the strength and modulus of the injection-molded parts, flexural tests were carried out. Flexural tests were conducted in accordance with ISO 178 using the universal testing machine specified above (MCT-2150) [17]. The loading speed was 10 mm/min with a span length of 40 mm. Single-gate molded specimens were tested in the TD. Flexural strength and modulus were calculated from the load–deflection curves. This test was performed five times under each condition. The mean and standard deviation of the calculated flexural strength and flexural modulus were found.

### 2.9. Notched Charpy Impact Test

Notched Charpy impact strength was evaluated as a means to assess the impact resistance of injection-molded parts. Notched Charpy impact strength was measured using a Charpy impact tester (Maiz Testing Machine, Kyoto, Japan) in accordance with ISO 179 [18]. The impact velocity was 2.91 m/s, with a 40 mm span length. A V-notch of approximately 1.0 mm depth was machined at the center of single-gate specimens, which were tested in the edgewise direction. This test was performed five times under each condition. The mean and standard deviation of the calculated notched Charpy impact strength were found.

### 2.10. Fourier Transform Infrared Spectroscopy

The extent of PP degradation was determined through the analysis of FT-IR spectra, which were acquired using a Fourier transform infrared spectrometer (Nicolet iS5, Thermo fisher Scientific K.K., Tokyo, Japan). The measurement method employed total reflection absorption (ATR), with 32 integration cycles, a wavenumber range of 4000–400 cm^−1^, and air as the background. Subsequent to the baseline correction of the obtained spectrum, the carbonyl index (CI) was calculated based on the specified area under band (SAUB) method [19]. Specifically, the CI was calculated using the following Equation (1), employing the absorbance of the peak corresponding to the carbonyl group (C=O) (approximately 1850–1650 cm^−1^) and the absorbance of the peak originating from the asymmetric bending vibration of the methylene group (CH_2_) (1500–1420 cm^−1^) as the reference peak.

(1) $$CI=\frac{Area\;under\;band\;1850-1650{\;cm}^{-1}}{Area\;under\;band\;1500-1420{\;cm}^{-1}}$$

This measurement was performed on three separate occasions, and the mean and standard deviation of CI were calculated for each instance.

## 3. Results

### 3.1. Changes in Melt Viscosity with Repeated Extrusion

**Figure 2:**
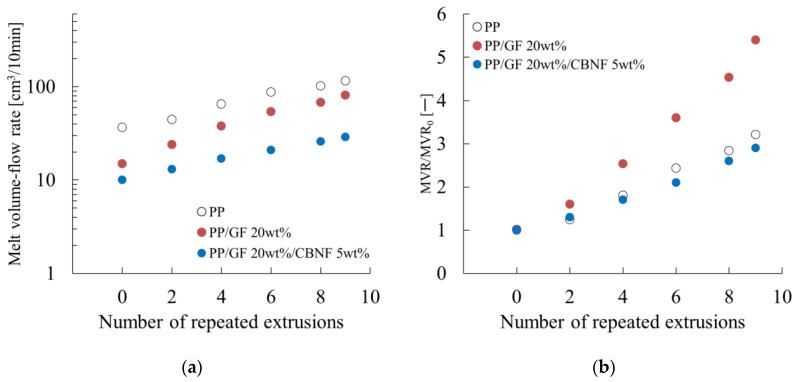
Repeated extrusion dependences of (**a**) the melt volume–flow rate (MVR) and (**b**) the relative MVR values (MVR/MVR_0_). MVR_0_ denotes the MVR when the number of repeated extrusions is zero.

Figure 2 shows the dependence of the melt volume-flow rate (MVR), as evaluated using a melt flow indexer, on the number of extrusion cycles. Figure 2a presents the MVR values of neat polypropylene (PP) and its composites with carbon nanofibers (CBNFs) and/or glass fibers (GFs) as a function of extrusion cycles. Measurements were conducted at 190 °C under a load of 2.160 kgf. The MVR values are shown on a logarithmic scale.

For neat PP, MVR increased gradually from an initial value of approximately 40 cm^3^/10 min to 100 cm^3^/10 min after nine extrusion cycles, corresponding to a 2.5-fold increase. The PP/GF composite showed intermediate behavior, with MVR increasing from 15 to 60 cm^3^/10 min. The presence of GF reduced initial melt flow because of increased viscosity, but its flow-restricting effect diminished after multiple processing cycles. The PP/GF/CBNF hybrid composite exhibited the lowest initial MVR of approx. 10 cm^3^/10 min, which increased to 25 cm^3^/10 min after ten cycles. The synergistic reinforcement of GF and CBNF contributed to the maintenance of the lowest flow rate throughout all cycles.

These results indicate that although all materials experienced processing-induced degradation, the incorporation of reinforcing fillers, particularly the hybrid system, stabilized melt viscosity effectively. This finding is crucially important for understanding the recyclability and processing window of these composites in industrial applications.

Figure 2b presents the relative MVR values (MVR/MVR_0_) normalized to the initial MVR for neat PP and the composites. All materials started at a normalized value of 1.0 at cycle 0.

The PP/GF composite exhibited the most pronounced increase, reaching 5.4 after nine cycles, representing a 5.4-fold rise. Neat PP showed a moderate increase, reaching 3.3 after nine cycles. The PP/GF/CBNF hybrid composite displayed an intermediate response, increasing to 2.9 after nine cycles. This result suggests that CBNFs partially suppress the degradation effects observed in the GF-only system through enhanced interfacial interactions and network formation.

The differences among the three formulations underscore the extremely important role of filler types and their combinations in determining processing stability. The hybrid system exhibits superior retention of rheological properties.

### 3.2. Changes in Glass Fiber Orientation and Length with Repeated Extrusion

**Figure 3:**
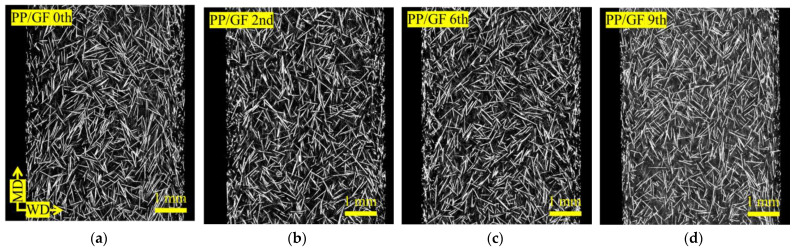

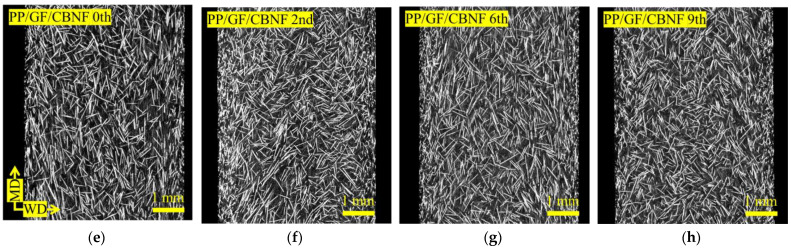
X-ray CT images of the skin region. Images (**a**–**d**) correspond to PP/GF, and images (**e**–**h**) to PP/GF/CBNF, after 0, 2, 6, and 9 extrusion cycles.

**Figure 4:**
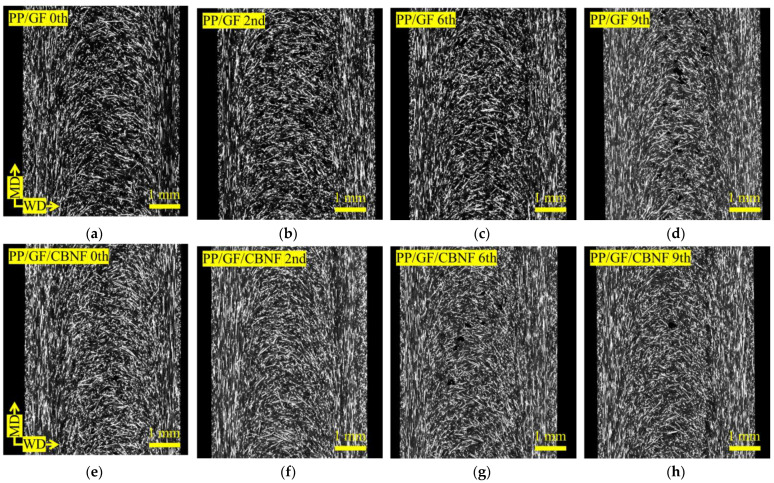
X-ray CT images of the core region. Images (**a**–**d**) correspond to PP/GF, and images (**e**–**h**) to PP/GF/CBNF, after 0, 2, 6, and 9 extrusion cycles.

Figure 3 shows X-ray CT images of the skin region, whereas Figure 4 shows those of the core region, as obtained using a microfocus X-ray CT system. Images (a–d) of Figure 3 and Figure 4 correspond to PP/GF, and images (e–h) of those figures to PP/GF/CBNF, after 0, 2, 6, and 9 extrusion cycles. In all cases, white fibrous structures representing GFs were dispersed randomly. Although visual changes in orientation and dispersion were observed with increasing extrusion cycles, precise interpretation was difficult from the images alone. Therefore, image analysis was conducted for quantification.

**Figure 5:**
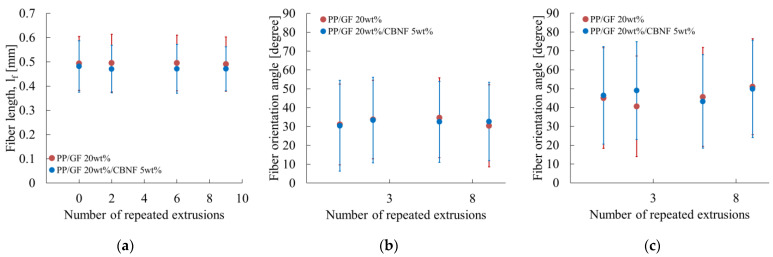
Repeated extrusion dependences of (**a**) average fiber length, (**b**) orientation angle in the skin region and (**c**) the angle in the core region.

Figure 5 exhibits the dependence of average fiber length and orientation angle, as determined from image analysis, on extrusion cycles. Figure 5a presents the average fiber length for PP/GF and PP/GF/CBNF. Error bars represent standard deviations. These values were derived from CT images of the skin region shown in Figure 3. The PP/GF composite maintained highly stable values, 0.47–0.49 mm, throughout the test period from an initial value of 0.49 mm. The PP/GF/CBNF hybrid also showed stable behavior, with a slight decrease from 0.48 mm initially to 0.47 mm after nine cycles.

Both composites showed error bars of ±0.10–0.12 mm, indicating uniform fiber length distributions. Even after nine cycles, both retained 96–98% of their initial fiber length, suggesting minimal fiber breakage. This excellent fiber length retention indicates that the chosen extrusion conditions prevented excessive fiber fracture effectively. The lack of any marked difference between PP/GF and the hybrid suggests that 5 wt% CBNF addition had little influence on the fracture mechanism of GFs.

Figure 5b shows the fiber orientation angle in the skin region. Here, the fiber orientation angle was defined relative to the MD. For PP/GF, the angle increased slightly from 31.5° initially to 34.0° after six cycles, then returned to 32.0° after nine cycles, remaining within a narrow range of 31.5–34.0°. The hybrid composite exhibited nearly identical behavior, ranging from 31.0° initially to 31.5° after nine cycles. Both systems showed large error bars (±18–22°), reflecting heterogeneous orientation in the injection-molded specimens.

The absence of any marked difference between the two composites indicates that CBNF addition (5 wt%) does not influence fiber orientation to any considerable degree. The small variation (<3°) through nine cycles suggests that orientation changes caused by repeated processing are minimal. The consistent average values despite large deviations highlight reproducible macroscopic orientation, contributing to predictable mechanical performance.

Figure 5c presents the fiber orientation angle in the core region. Particularly, PP/GF increased from 45.0° to 50.5° after two cycles. It then decreased to 41.0° after nine cycles. The hybrid exhibited a similar trend, decreasing slightly from 44.5° to 40.0°. Both showed very large error bars (±20–25°), reflecting strong heterogeneity because of fountain flow during injection molding [13]. Both systems showed slight decreases in orientation angle with increasing cycles, reaching ~40–41° after nine cycles, suggesting a gradual shift toward a flow-direction orientation. However, this change represented only a 4–5° decrease from the initial values, indicating overall stability.

### 3.3. Changes in Interfacial and Flexural Properties with Repeated Extrusion

**Figure 6:**
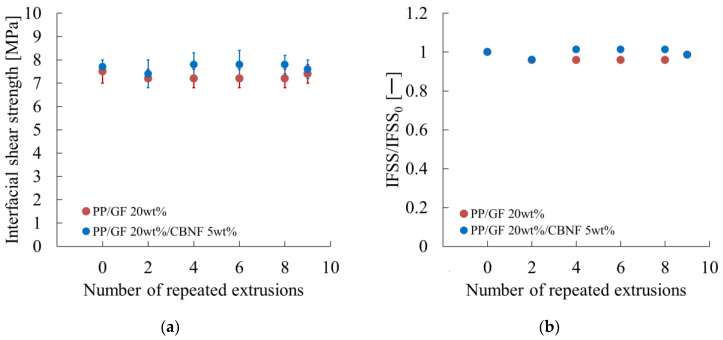
Repeated extrusion dependences of (**a**) the interfacial shear strength (IFSS) and (**b**) the relative IFSS values (IFSS/IFSS_0_). IFSS_0_ denotes the IFSS when the number of repeated extrusions is zero.

Figure 6 shows the interfacial shear strength (IFSS) obtained from short-beam shear tests. Figure 6a demonstrates that both PP/GF and the hybrid exhibited initial IFSS values of 7.5–7.7 MPa. Error bars represent standard deviations. The hybrid consistently maintained the highest IFSS across cycles, remaining between 7.4 and 7.8 MPa, even after nine cycles. After the PP/GF composite decreased slightly to 7.1 MPa after two cycles, it stabilized at approximately 7.4 MPa after nine cycles. No marked degradation was observed, confirming the excellent interfacial stability of both systems.

Figure 6b presents the normalized IFSS (IFSS/IFSS_0_). After PP/GF decreased slightly to 0.95 after two cycles, it recovered to 0.96–0.98 in subsequent cycles, probably because of realignment and removal of weak interfacial regions. The hybrid showed superior stability, with values of 0.96–1.02 throughout the cycles, suggesting that CBNFs formed protective interfacial layers that mitigated degradation. Both systems retained more than 95% of initial IFSS after nine cycles, confirming their suitability for recycling.

**Figure 7:**
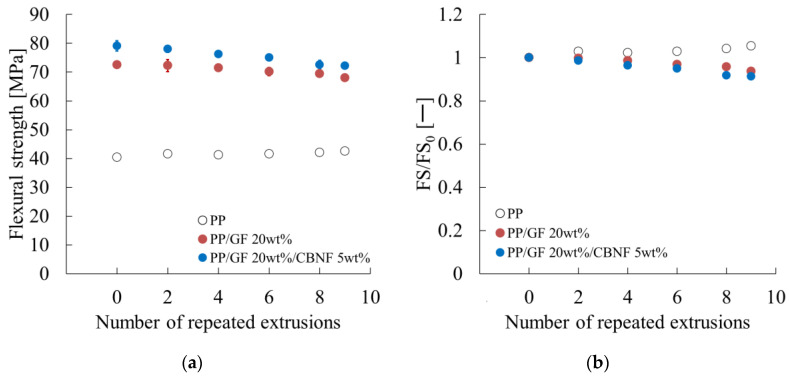
Repeated extrusion dependences of (**a**) the flexural strength (FS) and (**b**) the relative FS values (FS/FS_0_). FS_0_ denotes the FS when the number of repeated extrusions is zero.

Figure 7 shows the dependence of bending strength on extrusion cycles obtained from the results of the three-point bending test. Figure 7a shows the flexural strength. Error bars represent standard deviations. The hybrid exhibited the highest values, decreasing gradually from 78.5 MPa initially to 72.0 MPa after nine cycles, retaining 92% of the initial value. The PP/GF composite decreased from 72.5 to 67.5 MPa, retaining 93%. Neat PP exhibited the lowest values, remaining nearly constant at approx. 40 MPa.

Figure 7b shows normalized flexural strength. Both composites decreased gradually, with the hybrid maintaining high absolute values despite a 92% relative retention. Neat PP exhibited a slight increase because of densification effects.

**Figure 8:**
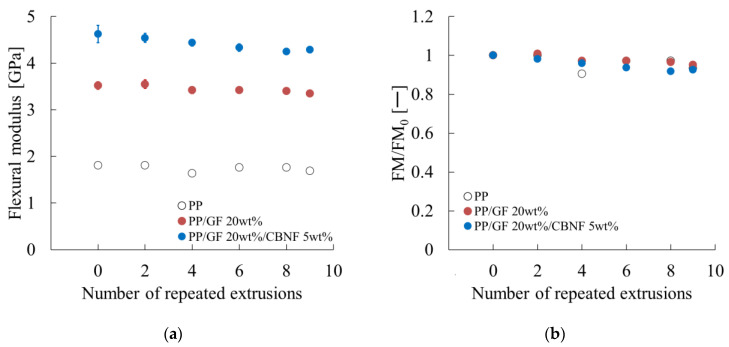
Repeated extrusion dependences of (**a**) the flexural modulus (FM) and (**b**) the relative FM values (FM/FM_0_). FM_0_ denotes the FS when the number of repeated extrusions is zero.

Figure 8 presents the dependence of the flexural modulus on extrusion cycles obtained from the results of the three-point bending test. Figure 8a shows the flexural modulus. Error bars represent standard deviations. The hybrid maintained the highest modulus, decreasing slightly from 4.55 to 4.30 GPa after nine cycles (95% retention). PP/GF remained stable at approx. 3.35 GPa after nine cycles. Neat PP showed the lowest modulus (approx. 1.70 GPa).

Figure 8b shows normalized modulus values. All materials retained more than 94% of their initial modulus after nine cycles, indicating excellent structural stability, with the hybrid exhibiting the best overall performance.

### 3.4. Changes in Notched Charpy Impact Strength with Repeated Extrusion

**Figure 9:**
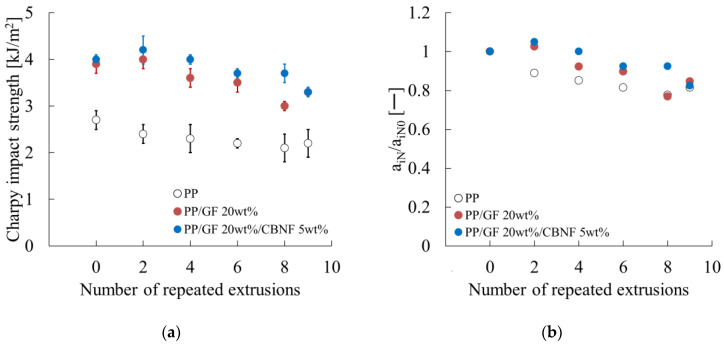
Repeated extrusion dependences of (**a**) the Charpy impact strength (a_iN_) and (**b**) the relative a_iN_ values (a_iN_/a_iN0_). a_iN0_ denotes the a_iN_ when the number of repeated extrusions is zero.

Figure 9 presents the notched Charpy impact strength. Error bars represent standard deviations. Figure 9a shows that the hybrid exhibited the highest initial value (4.00 kJ/m^2^), which increased slightly to 4.20 kJ/m^2^ after two cycles, then decreased gradually to 3.35 kJ/m^2^ after nine cycles, retaining 84%. The PP/GF composite showed a similar trend, decreasing significantly after eight cycles. Neat PP exhibited the lowest strength, decreasing steadily because of embrittlement from molecular weight reduction.

Figure 9b portrays normalized values. The hybrid increased to 1.05 after two cycles, then decreased to 0.84 after nine cycles. PP/GF decreased to 0.77 after eight cycles, then recovered slightly. Neat PP decreased gradually to 0.85 after nine cycles.

These results indicate that impact strength deteriorated as a result of repeated extrusion, but the hybrid composite retained superior absolute and relative values, demonstrating CBNF effectiveness for stabilizing impact performance during recycling.

## 4. Discussion

### 4.1. Mechanism of Improved Processing Stability by Carbon Nanofiber Addition

The CBNF-containing system exhibited superior melt viscosity stability, as shown in Figure 2. This behavior is explainable by two major mechanisms.

First, for neat PP, radicals are generated under heat and shear during repeated extrusion. These radicals reduce molecular weight and stereoregularity, which in turn decreases melt viscosity [20]. Radicals are generated particularly under localized overheating during shear. CBNFs, because of their intrinsic thermal conductivity, can suppress such localized overheating, thereby reducing radical formation. Consequently, molecular weight degradation of PP is suppressed. Moreover, melt viscosity is retained.

**Figure 10:**
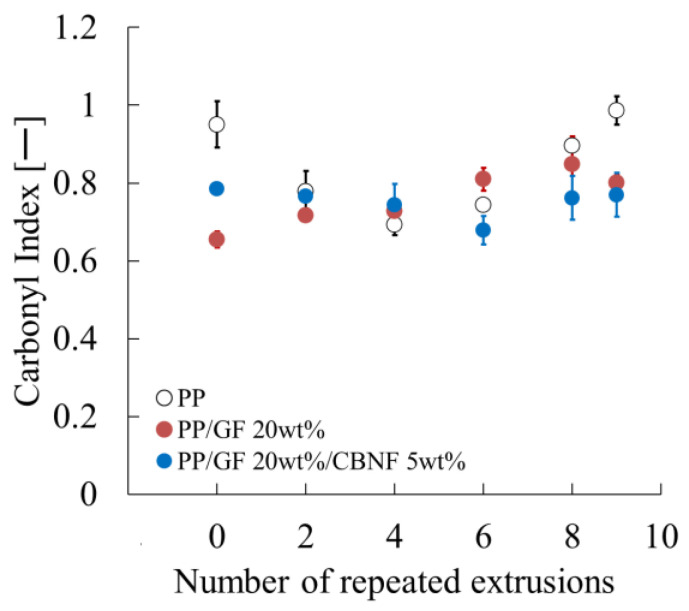
Carbonyl index evaluation results for each composition.

In order to verify the aforementioned hypothesis, the present study calculated the carbonyl index (CI) using Fourier transform infrared spectroscopy (FT-IR) and evaluated its dependence on the number of extrusion cycles. As illustrated in Figure 10, the CI evaluation results for each composition are presented. The findings indicate a strong correlation between the behavior of the CI and its composition.

In the preliminary stage, which is the zero repeated extrusion, PP exhibited the highest CI value, with twin-screw extrusion being the sole extrusion process employed. Subsequently, the CI decreased until the number of extrusions reached four, after which it began to increase. The elevated CI value recorded at the preliminary stage can be ascribed to the vigorous kneading action occurring during twin-screw extrusion, concomitant with the entrapment of air [21]. Twin-screw extruders generate stronger shear stress compared to single-screw extruders, which makes them more prone to entraining air during the molding process. It is hypothesized that oxidation reactions were promoted by entrained air, and the resulting increase in oxidation products containing carbonyl groups led to the apparently high CI at the preliminary stage. Consequently, during the single-screw extrusion process, the kneading action is relatively mild, leading to reduced air entrainment. The oxidation products generated during twin-screw extrusion (low molecular weight components, peroxides, etc.) were likely removed through thermal decomposition or volatilization, causing the CI to decrease up to the fourth repeated extrusion cycle. As indicated by the results of the study, from this point onwards, the thermal oxidation reaction occurring during single-screw extrusion became dominant, leading to an increase in the CI.

In the PP/GF system, with the incorporation of 20 wt% glass fiber into the PP, the substantial increase in CI noted at the zero repeated extrusion for pure PP was not observed. Conversely, the CI exhibited a consistent increase with the number of extrusion cycles. Glass fibers have been shown to exhibit higher thermal conductivity in comparison to polypropylene (PP), and they also demonstrate a capacity to disperse shear stress at the fiber interface. This suppression led to a subsequent inhibition of PP molecular chain scission and radical generation, consequently impeding carbonyl group formation. Consequently, the CI increase during the twin-screw extrusion stage was minimal. The increase in the critical temperature, i.e., the critical polymer concentration (CI), associated with repeated extrusion is presumed to result from the progression of localized overheating at the fiber-polymer interface, in addition to the thermal oxidation reaction similar to that observed in pure polypropylene (PP).

Conversely, in the system where 5 wt% CBNF was incorporated into PP/GF 20 wt%, the CI at the zero repeated extrusion exhibited a higher value compared to the PP/GF system. However, the CI remained nearly constant, even after multiple extrusion cycles. The elevated CI value at the zero repeated extrusion is hypothesized to be attributable to the concurrent detection of carbonyl groups that are inherently present on the CBNF surface. The rationale behind the absence of change in CI, despite an augmentation in extrusion cycles, is probably attributable to the elevated thermal conductivity exhibited by CBNF. This approach effectively diffuses localized temperature peaks generated at fiber ends and friction surfaces during extrusion throughout the system, thereby suppressing thermal decomposition and oxidation caused by high temperatures. The suppression of localized temperature increases also hindered radical generation and peroxide decomposition, ultimately inhibiting CI increase.

Second, CBNFs interact strongly with PP chains. Because CBNFs have a submicrometer diameter, their specific interfacial surface area is orders of magnitude larger than that of GFs. As a result, PP chains at the CBNF interface are constrained considerably. Even when the PP matrix undergoes molecular weight degradation, this constraint effect stabilizes the overall viscosity of the system.

Chemical interactions further reinforce this effect. Earlier work has demonstrated that MAH-PP can promote covalent and hydrogen bonding between carbon fibers and PP [22,23]. A similar mechanism is expected for CBNFs. Possible covalent interactions might include Diels–Alder reactions between maleic anhydride and conjugated carbon surfaces, as well as radical reactions at defect or edge sites [24]. Hydrogen bonding via C=O groups of maleic anhydride and water molecules [25] could also occur, although it is unlikely to dominate under melt processing because of desorption at high temperatures. Direct experimentally obtained evidence was not obtained from this study, but these mechanisms represent plausible explanations. Covalent bonding is tentatively considered as the main interfacial interaction between CBNF and MAH-PP.

Through such covalent bonds, CBNFs are coupled strongly with MAH-PP. Consequently, PP chains surrounding MAH-PP are indirectly constrained. Given the submicrometer scale and high aspect ratio of CBNFs, numerous PP chains are restricted at the CBNF surface. This restriction persists even after repeated extrusion because covalent bonds are not broken easily. Therefore, the chemical interaction at CBNF surfaces limits PP chain mobility effectively and explains the superior melt viscosity stability of CBNF-containing systems.

### 4.2. Mechanism of Flexural Property Degradation Under Repeated Extrusion

Figure 7 and Figure 8, respectively, present the dependence of flexural strength and modulus on repeated extrusion. The degradation mechanism was analyzed in terms of fiber-related and matrix-related factors.

Fiber-related parameters remained almost unchanged. As shown in Figure 6a, the variation in interfacial shear strength was negligible. Fiber length and orientation angle in the skin region also showed only small changes, as shown in Figure 5a,b. These results indicate that geometric contributions from fibers were influenced only slightly by extrusion.

Therefore, the observed degradation in flexural properties can be attributed mainly to PP matrix degradation. Takayama et al. [8] reported that shear and heat during extrusion generate radicals, which disrupt stereoregularity and reduce crystallinity. These effects increase free volume and engender reduced yield stress and tensile modulus, while increasing Poisson’s ratio [26]. A similar mechanism is likely to occur in both PP/GF and PP/GF/CBNF composites, suggesting that radical-induced PP degradation is the main cause of flexural property loss.

**Figure 11:**
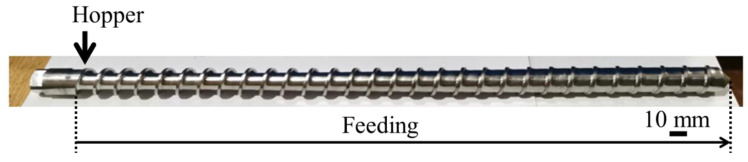
Overview of the full-flight screw used in a single-screw extruder.

The limited change in fiber length might be explained by the screw geometry of the single-screw extruder used for this study. The single-screw extruder utilized in this study employs a full-flight screw as shown in Figure 11. This screw exhibits a continuous flight profile over its entire length, devoid of any mixing elements or barrier structures within the kneading section. Consequently, this design leads to comparatively low shear stress levels during the extrusion process. In the context of extrusion molding of fiber-reinforced thermoplastic resins, it has been established that the primary factors inducing fiber breakage are the shear stress and tensile stress generated by screw rotation. In regions of high shear, such as the mixing section, it has been established that fibers undergo fragmentation due to the interactions between fibers and the mechanical contact with the screw and barrel. This phenomenon results in a substantial decrease in the average fiber length [27].

Conversely, in the full-flight screw, the melting and conveying process of the resin progresses relatively laminar, thereby suppressing the formation of localized high-shear zones. This phenomenon has the effect of reducing the frequency of fiber collisions within the molten resin. It also mitigates compressive stress within the screw grooves and friction contact with the barrel wall. Consequently, the overall mechanical load on the fibers is reduced, and the progression of breakage is suppressed. In light of the aforementioned findings, it can be posited that the fiber length retention observed in this study is attributable to the reduction in shear stress during the extrusion process achieved by the full-flight screw.

These findings highlight an important implication for mechanical recycling: even if fiber length is preserved, degradation of the PP matrix alone can reduce flexural properties considerably.

### 4.3. Mechanism of Notched Impact Strength Degradation Under Repeated Extrusion

This subsection presents a discussion of the dependence of notched Charpy impact strength on repeated extrusion, as presented in Figure 9.

First, the changes in fiber-related parameters were examined. As portrayed in Figure 6a, the change in interfacial shear strength was negligible. Figure 5a,c further revealed that the change in fiber length was small, whereas the fiber orientation angle in the core region tended to decrease concomitantly with increasing extrusion cycles.

The relation between these changes and notched Charpy impact strength can be interpreted using the mechanical model proposed by Jiang et al. [28]. They suggested that the energy dissipation during notched Charpy impact testing is converted mainly into frictional energy arising from fiber pull-out. They proposed the following Equation (2) [28].

(2) $$a_{iN}=\tau_{i}\cos\varphi S_{f}l_{p}^{2}$$

In that equation, τ_i_ denotes the interfacial shear strength, φ is the average orientation angle in the core region excluding the shear orientation zone, S_f_ represents the specific interfacial surface area, and l_p_ stands for the pull-out length. φ is defined relative to the WD. For cylindrical fibers, S_f_ can be expressed as Equation (3).

(3) $$S_{f}=\frac{4V_{f}}{d}$$

Therein, d stands for the fiber diameter; V_f_ signifies the fiber volume fraction. Moreover, l_p_ is classifiable into two cases depending on the relation between the critical fiber length l_c_ and the remaining fiber length l_f_, as expressed in Equation (4) [10].

(4) $$l_{p}=\left\{\begin{array}{c} \frac{l_{f}}{2}\;\mathrm{for}\;l_{c}>l_{f}\\ \frac{l_{c}}{2}\;\mathrm{for}\;l_{c}<l_{f} \end{array}\right.$$

**Figure 12:**
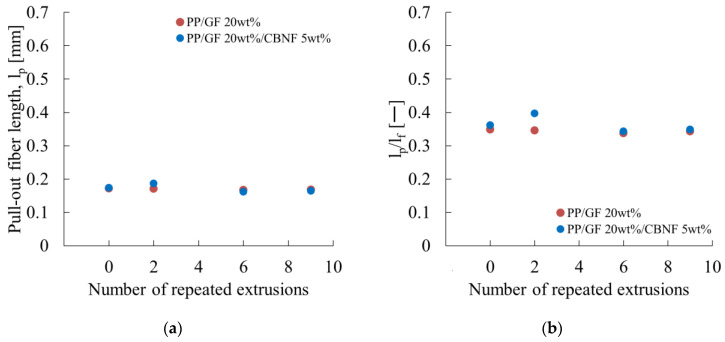
Repeated extrusion dependences of (**a**) the pull-out fiber length (l_p_) and (**b**) the ratio of l_p_ to l_f_ (l_p_/l_f_).

First, l_p_ was calculated by substituting the experimentally obtained results into Equation (1). Then the relation with l_f_ was examined. Figure 12 portrays the calculation results.

Figure 12a presents the pull-out fiber length as a function of extrusion cycles for PP/GF and PP/GF/CBNF composites. Both composites exhibited initial values of approximately 0.175 mm. For PP/GF, l_p_ changed to 0.180 mm after two cycles, 0.165 mm after six cycles, and 0.170 mm after nine cycles, remaining stable within a narrow range of 0.165–0.180 mm. The hybrid composite also exhibited stable behavior, with l_p_ changing from 0.175 mm initially to 0.178 mm after two cycles, 0.165 mm after six cycles, and 0.168 mm after nine cycles. The absence of significant differences between the two composites indicates that CBNF addition does not alter the interfacial fracture mechanism to any considerable degree.

Figure 12b shows the ratio of l_p_ to l_f_ as a function of extrusion cycles. When the remaining fiber length is shorter than l_c_, Equation (3) gives a ratio of 0.5. For PP/GF, the ratio varied within a narrow range of 0.34–0.38, whereas the hybrid composite showed similar behavior (0.34–0.40). Ratios smaller than 0.5 indicate that l_f_ is longer than l_c_.

**Figure 13:**
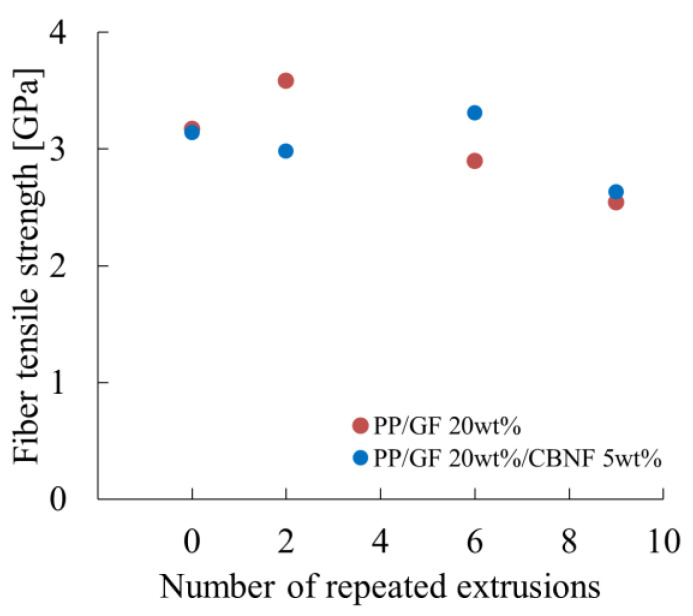
Dependences of fiber tensile strength on extrusion cycles.

Takayama et al. proposed an equation for l_c_ based on the Kelly–Tyson model with additional consideration of fiber orientation angle expressed as Equation (4) as [29]

(5) $$l_{c}=\frac{d\sigma_{F0}\sin^{2}\varphi }{8\tau_{i}}$$

where σ_F0_ denotes the tensile strength of the fiber in the axial direction. Using this equation, σ_F0_ was estimated. Figure 13 portrays the dependence of fiber tensile strength on extrusion cycles. For PP/GF, σ_F0_ increased considerably from 3.25 GPa initially to 3.70 GPa after two cycles, then decreased stepwise to 3.00 GPa after six cycles and 2.60 GPa after nine cycles. For the hybrid composite, σ_F0_ changed from 3.20 GPa initially to 3.10 GPa after two cycles, 3.50 GPa after six cycles, and 2.70 GPa after nine cycles, showing a characteristic peak at six cycles.

The increase or retention of strength after two cycles in both composites suggest surface cleaning of fibers or selective removal of weak fibers. However, because no direct surface characterization was conducted in this study, this explanation remains hypothetical and should be verified in future work. In the GF-only system, the marked increase after two cycles might be attributable to the redistribution or activation of surface treatments. By contrast, the hybrid composite showing a peak at six cycles might imply that CBNFs help alleviate stress concentration at fiber surfaces, which could contribute to strength retention. Further microstructural evidence is expected to be necessary to confirm this mechanism. The decrease to 2.60–2.70 GPa after nine cycles reflects fatigue degradation of fibers under repeated thermo-mechanical stress [30,31]. The strength reduction rate from initial to nine cycles was 20% for PP/GF and 16% for the hybrid, indicating that CBNF addition slightly suppresses fiber degradation.

### 4.4. Recyclability and Practical Challenges of PP/GF/CBNF Hybrid Composites

Repeated extrusion experiments demonstrated that PP/GF/CBNF hybrid composites possess excellent recyclability. Even after nine extrusion cycles, retention rates of 92% for flexural strength, 95% for flexural modulus, and over 95% for interfacial shear strength were achieved. Such high retention rates constitute strong evidence underscoring the potential of CBNFs for use in sustainable composites.

The outstanding thermal and chemical stability of CBNFs are a salient benefit. Their covalent carbon network remains intact at PP processing temperatures (190–230 °C), ensuring structural stability [32]. Moreover, their high thermal conductivity (10–100 W/m·K) prevents localized overheating, leading to uniform temperature distribution and reducing heterogeneity in thermal degradation [33]. This degradation contributes to stable recyclability and to improved reliability of recycled materials.

The major limitation of CBNFs is their cost. High-quality CBNFs are currently far more expensive than GFs [34]. The manufacturing cost of PP/GF is low even compared to other FRTPs, and the cost advantage of mechanically recycling this material is minimal [35]. However, the implementation of PP/GF/CBNF results in an increase in the cost of new material. Consequently, a relative cost advantage is expected to emerge for the application of recycled material. In addition, this study demonstrated that the addition of only 5 wt% CBNF markedly improved both initial properties (8% increase in flexural strength, 30% increase in modulus) and property retention after recycling. Such durability offers potential life-cycle cost benefits, particularly in automotive applications, for which weight reduction and recyclability are crucially important. With growing emphasis on circular economy regulations in Europe, recyclable composites are expected to become increasingly important.

CBNF dispersion also played an important role. Using MAH-PP as a compatibilizer enhanced CBNF dispersion through covalent bonding at the CBNF surface. This bonding suppressed CBNF agglomeration and remained stable during repeated extrusion, thereby maintaining mechanical performance.

Nevertheless, the environmental effects of CBNFs must be evaluated carefully. Their production often requires high-temperature processes (approx. 1000 °C), leading to considerably high degrees of energy consumption and CO_2_ emissions [36,37]. From a life-cycle assessment perspective, however, their superior recyclability and durability might offset these effects. The potential toxicity of CBNFs also requires continuous monitoring. The extant research has demonstrated that carbon nanofibers induce substantial oxidative stress and genetic-level changes in freshwater algae, exhibiting toxicity even in the absence of other stressors. In the context of nanoplastics, there is a growing body of literature pointing to a synergistic enhancement in the toxicity of the nanoplastics themselves. This enhancement in toxicity has been observed to result in the inhibition of algal growth, manifesting through the impairment of cell membranes, an increase in ROS production, and abnormalities in energy metabolism [38]. It is imperative to acknowledge the pivotal roles that oxidative stress responses, glucose metabolism, and energy metabolism have been shown to play in the context of particle-induced toxicity. To comprehensively grasp the distinctive biological effects of nanomaterials, it is essential to engage in long-term observation.

Finally, because CBNFs are carbon-based, they are recyclable, theoretically, as carbon sources. Recovery of carbon from used CBNF composites could be employed as feedstock for future CBNF production, thereby contributing to a closed-loop carbon recycling system.

## 5. Conclusions

This study developed hybrid composites of glass fiber-reinforced polypropylene (PP/GF) containing carbon nanofibers (CBNFs) to improve their mechanical recyclability. The effects of repeated extrusion on the physical and mechanical properties were examined systematically.

The PP/GF 20 wt%/CBNF 5 wt% hybrid composite demonstrated superior melt viscosity stability, limiting the increase in melt volume rate (MVR) to 2.9 times after nine extrusion cycles, compared with a 5.4-fold increase in PP/GF. This superior melt viscosity stability was attributed to the suppression of localized overheating by the high thermal conductivity of CBNFs and the restriction of PP chains through covalent bonding with MAH-PP. The average glass fiber length remained stable, retaining 96–98% of the initial length after nine cycles because of the low-shear conditions of the full-flight screw.

The hybrid composite exhibited 8% higher flexural strength and 30% higher flexural modulus than PP/GF at the initial state. After nine cycles, it retained 92% of flexural strength, 95% of flexural modulus, >95% of interfacial shear strength, and 84% of notched Charpy impact strength. The decrease in impact strength was attributed mainly to glass fiber degradation and matrix viscosity reduction, while CBNF dispersion showed little effect.

Overall, the PP/GF/CBNF hybrid composite demonstrated excellent durability against repeated processing, highlighting CBNF as a promising reinforcement for recyclable thermoplastics. A comparison of manufacturing costs reveals an indisputable increase when PP/GF is taken into consideration. However, this increase also renders the material highly suitable for the application of recycled materials. These findings provide practical guidelines for designing lightweight, recyclable composites to meet the needs of automotive and transportation applications and to meet the increasing needs of circular economy development.

## Figures and Tables

**Table 1:** Injection molding conditions.

PP [wt%]	GF [wt%]	MAH-PP [wt%]	CBNF [wt%]	*T*_inj _ [°C]	*T*_mold _ [°C]	*V*_inj _ [m/s]	*P*_hold _ [MPa]	*T*_inj _ [s]	*T*_cool _ [s]
77	20	3	-	230	50	30	56	10	15
72	20	3	5	230	50	30	70	10	15
100	-	-	-	230	50	30	70	10	15

## Data Availability

The original contributions presented in this study are included in the article. Further inquiries can be directed to the corresponding author.
